# A recognition model for winter peach fruits based on improved ResNet and multi-scale feature fusion

**DOI:** 10.3389/fpls.2025.1545216

**Published:** 2025-04-09

**Authors:** Yan Li, Chunping Li, Tingting Zhu, Shurong Zhang, Li Liu, Zhanpeng Guan

**Affiliations:** Faculty of Megadata and Computing, Guangdong Baiyun University, Guangzhou, China

**Keywords:** ResNet, peach, object detection, deep learning, BiFPN

## Abstract

With the continuous advancement of modern agricultural technologies, the demand for precision fruit-picking techniques has been increasing. This study addresses the challenge of accurate recognition and harvesting of winter peaches by proposing a novel recognition model based on the residual network (ResNet) architecture—WinterPeachNet—aimed at enhancing the accuracy and efficiency of winter peach detection, even in resource-constrained environments. The WinterPeachNet model achieves a comprehensive improvement in network performance by integrating depthwise separable inverted bottleneck ResNet (DIBResNet), bidirectional feature pyramid network (BiFPN) structure, GhostConv module, and the YOLOv11 detection head (v11detect). The DIBResNet module, based on the ResNet architecture, introduces an inverted bottleneck structure and depthwise separable convolution technology, enhancing the depth and quality of feature extraction while effectively reducing the model’s computational complexity. The GhostConv module further improves detection accuracy by reducing the number of convolution kernels. Additionally, the BiFPN structure strengthens the model’s ability to detect objects of different sizes by fusing multi-scale feature information. The introduction of v11detect further optimizes object localization accuracy. The results show that the WinterPeachNet model achieves excellent performance in the winter peach detection task, with P = 0.996, R = 0.996, mAP50 = 0.995, and mAP50-95 = 0.964, demonstrating the model’s efficiency and accuracy in the winter peach detection task. The high efficiency of the WinterPeachNet model makes it highly adaptable in resource-constrained environments, enabling effective object detection at a relatively low computational cost.

## 1 Introduction

In modern agricultural production, fruit harvesting is a critical process that not only affects the freshness and quality of fruits but also directly impacts the economic benefits for farmers (Li et al., 2022b; Wang et al., 2024; Zhang et al., 2024). With the growth of the global population and changes in consumption patterns, the demand for higher fruit yield and quality has been steadily increasing. Traditional manual harvesting methods can no longer meet the requirements of modern, efficient, and precise agriculture (Shi et al., 2024b; Soliman et al., 2012). Consequently, automated and intelligent harvesting technologies have become a key focus of research in the agricultural field. Among various fruits, peaches are particularly significant due to their widespread popularity, making research on peach harvesting techniques highly relevant in practical applications.

Peach harvesting requires not only high efficiency but also minimal damage to the fruit during the process to maintain its freshness and appearance. However, the complex growth environment of peach trees, the diversity of fruit shapes, and the richness of color variations pose numerous challenges for traditional machine vision technologies in peach recognition and localization (Chaivivatrakul and Dailey, 2014; Cui et al., 2022). These challenges include, but are not limited to, variations in lighting conditions, mutual occlusion between fruits and branches or leaves, and inconsistencies in fruit maturity (Tang et al., 2023). These factors complicate automated peach harvesting, limiting the applicability and efficiency of harvesting robots.

In recent years, the development of deep learning technologies, particularly breakthroughs in convolutional neural networks for image recognition, has provided new solutions to these challenges (Fan et al., 2022; Li, 2022; Traore et al., 2018). Object detection algorithms in deep learning are mainly categorized into two-stage detection algorithms and single-stage detection algorithms. Two-stage detection algorithms explicitly propose candidate regions and transform the detection task into a classification problem for these regions. The R-CNN series (R-CNN, Fast R-CNN, Faster R-CNN, etc.) are representative examples. R-CNN extracts candidate regions from an image using selective search, then uses CNNs to extract features and classify them (Girshick et al., 2014). While R-CNN achieved breakthroughs in accuracy, its need to extract features and classify each candidate region individually resulted in low speed and efficiency (Mijwil et al., 2022). Fast R-CNN addressed the efficiency issues of R-CNN by introducing ROI pooling and a multi-task loss function, significantly improving detection speed and accuracy (Wi et al., 2024). Faster R-CNN further proposed the region proposal network, which automatically generates region proposals and shares the feature extraction process with the detection network, greatly enhancing detection performance and speed (Ren et al., 2016; Xiao et al., 2020).

Single-stage detection algorithms, on the other hand, skip the region proposal step and directly transform object detection into a regression problem, offering the advantages of higher speed and efficiency (Liu and Dong, 2024; Xiao et al., 2024). Representative algorithms include the You only look once (YOLO) series (Alif and Hussain, 2024) and the SSD series (Liu et al., 2016). The YOLO series, in particular, has gained wide attention for its end-to-end efficient detection. From YOLOv1 to YOLOv3, the introduction of anchor boxes and multi-scale feature fusion significantly improved the ability to detect small objects. YOLOv4 through YOLOv7 further balanced speed and accuracy and enhanced adaptability to complex scenarios through cross stage partial structures, path aggregation networks, and task decoupling mechanisms. The latest iterations, YOLOv8 through YOLOv11, have focused on lightweight design, multi-source data fusion, and robustness in complex scenarios, further improving efficiency and practicality in real-world applications. The YOLO series algorithms are especially suitable for scenarios requiring high real-time performance. This study addresses the challenges of winter peach recognition in complex environments, such as variations in lighting conditions, occlusion, and inconsistent levels of stacking. Based on the YOLO framework, a specialized object detection model for winter peach detection was designed to enhance the accuracy of fruit localization for harvesting robots. The YOLO algorithm divides the network structure into three parts: the backbone, neck, and head networks. The backbone network is responsible for extracting basic features from the image, the neck network enhances multi-scale feature representation through feature fusion, and the head network completes object classification and localization tasks.

In object detection tasks, the backbone network, as the core feature extraction module, directly impacts the overall detection performance. Common backbone networks include visual geometry group (VGG), residual network (ResNet), the MobileNet series, EfficientNet, and CSPDarknet. VGG is renowned for its simple network structure and stacked convolutional layers, offering strong feature extraction capabilities but with high parameter and computation costs (Rahman et al., 2024). ResNet introduced residual structures to effectively mitigate the vanishing gradient problem, enabling deeper network training with strong feature representation capabilities and widespread applicability (Islam et al., 2023; Yang et al., 2024). MobileNet, centered on depthwise separable convolution (DWConv), significantly reduces parameter and computational costs, making it a typical example of lightweight models (Xu et al., 2022). EfficientNet uses a compound scaling strategy to achieve an excellent balance between model depth, width, and resolution, though at a higher computational cost (Jin et al., 2023). CSPDarknet, widely adopted in YOLOv4 and later versions, optimizes feature learning capability and efficiency through gradient branching (Jia et al., 2023). After comparison, we selected ResNet as the backbone network due to its balance between feature representation capability and computational cost. Its residual structure not only enhances the stability of network training but also provides high generalizability, meeting the requirements of our object detection tasks.

The neck structure is responsible for fusing and enhancing the multi-scale features extracted by the backbone network, thereby improving the detection capability for targets of varying sizes. Common neck structures include feature pyramid network (FPN), path aggregation network (PANet), and bidirectional feature pyramid network (BiFPN). FPN enhances the detection of small objects through a top-down feature fusion mechanism (Karthikeyan et al., 2024). PANet builds upon this by adding a bottom-up path aggregation module, further improving the representation of multi-scale features (Lawal, 2024). BiFPN combines weighted feature fusion and bidirectional feature flow mechanisms, effectively increasing feature utilization and achieving greater computational efficiency through node weight optimization (Li et al., 2022a; Zhang et al., 2023). Considering the balance between feature fusion effectiveness and computational cost, this study selects BiFPN as the neck structure of the model to fully exploit multi-scale information while meeting the lightweight requirements of the model.

The detection head in single-stage object detection networks is a critical module that directly performs object classification and bounding box regression. Its structure significantly affects detection precision and efficiency. Anchor-free detection heads provide a more direct and flexible approach to object detection by reducing dependency on predefined anchor boxes. This approach offers clear advantages in terms of detection speed and adaptability. Anchor-free detection heads predict the bounding boxes of objects directly from image features instead of relying on a predefined set of anchor boxes, simplifying the detection process and reducing computational complexity (Xu et al., 2024; Zhao et al., 2023). The latest YOLOv11 detection head (v11detect) employs an anchor-free design and incorporates DWConv, improving processing speed while maintaining high precision (He et al., 2024). Consequently, this study adopts the v11detect as the detection head structure.

To improve the efficiency of automated winter peach recognition, this study developed a winter peach recognition and monitoring model, WinterPeachNet, based on an improved ResNet architecture. The model aims to achieve efficient and accurate recognition of winter peaches by optimizing the network structure. The core backbone network of the model is based on the depthwise separable inverted bottleneck ResNet (DIBResNet) structure, with the BiFPN architecture and GhostConv module introduced in the neck network. The head network utilizes the v11detect structure. Through these improvements, the model is designed to meet the real-time and accuracy requirements for automated winter peach recognition, providing an efficient and reliable technological solution for winter peach harvesting. This, in turn, contributes to the development and application of automation technology in winter peach harvesting.

## 2 Materials and methods

### 2.1 Dataset construction

**Figure 1:**
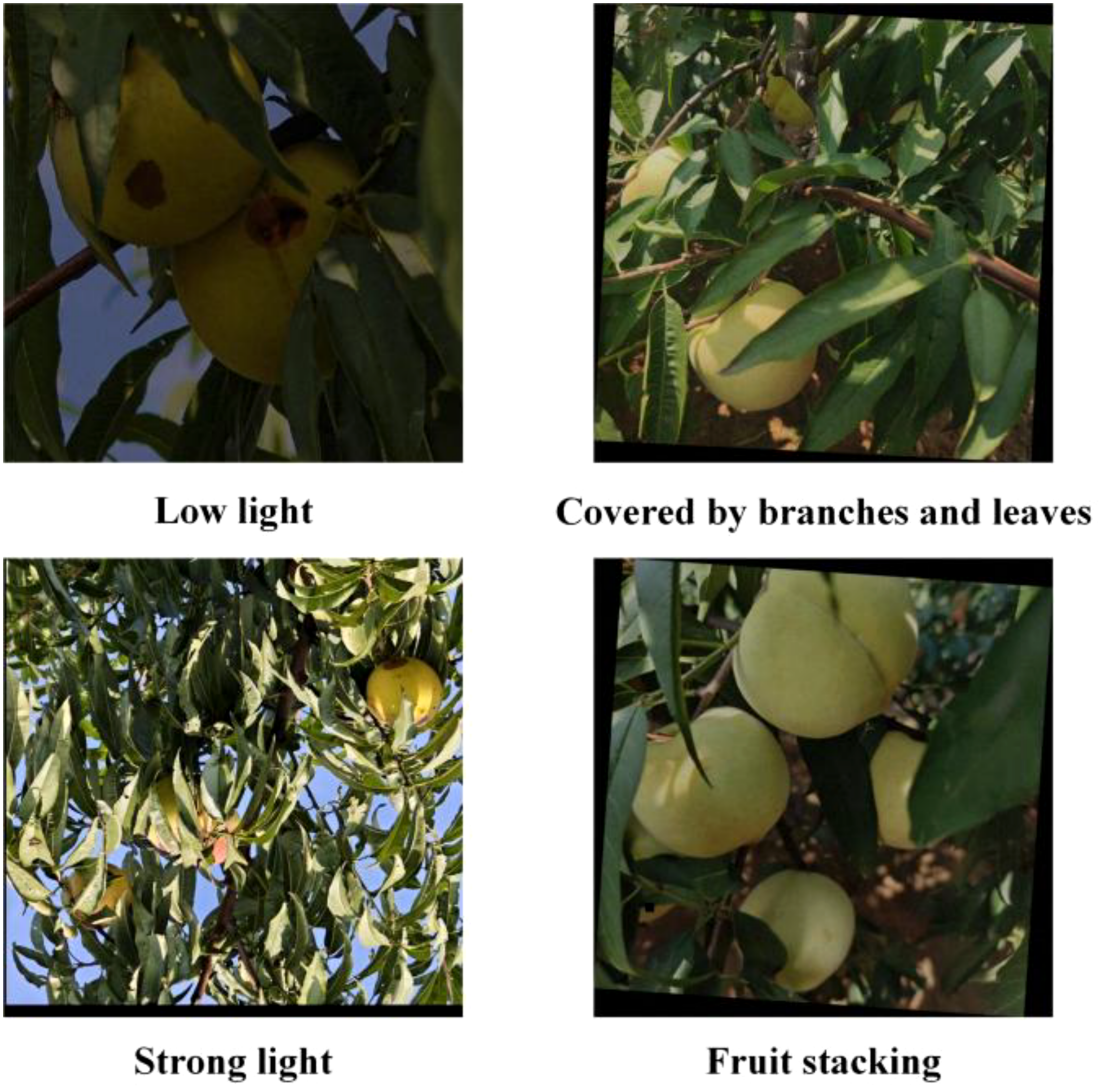
Sample images of winter peaches.

In this study, the iQoo 12 smartphone was used to collect winter peach data. The device features a multi-camera setup, with the main camera offering a 50-megapixel resolution and supporting optical image stabilization, enabling it to accurately capture detailed images and color information. Additionally, the device’s advanced image processing technology optimizes image quality, ensuring the accuracy and reliability of the data. The data collection site is located in an orchard in Luoyang, Henan Province, China. The data was collected on October 6, 2024. The image resolution is 3072 × 3072. To enhance the diversity of the dataset and improve the model’s generalization ability, advanced data augmentation techniques were employed, including but not limited to image rotation, scaling, and color adjustment to simulate various environmental conditions and lighting changes. Furthermore, to ensure precise and consistent data annotation, LabelImg was selected as the annotation tool. Its user-friendly interface and efficient annotation workflow greatly facilitated accurate labeling. The final dataset used for training consists of 1,250 images, which cover a variety of scenarios, including different lighting conditions, branch and leaf occlusion, and fruit stacking, providing diverse feature information. The dataset was split into training, testing, and validation sets in an 8:1:1 ratio, with 877, 187, and 186 images in each set, respectively. **Figure 1** presents a sample of the winter peach images included in the dataset.

### 2.2 Construction of the winter peach detection model

#### 2.2.1 DIBResNet

In this study, the ResNet was adopted as the core architecture for image recognition. By introducing a residual learning mechanism, ResNet effectively addresses the vanishing gradient problem in deep network training, making it feasible to construct deeper network structures. This network comprises a series of residual blocks, each containing multiple convolutional layers and a residual connection. The residual connection allows the input to bypass one or more layers and be added to the output, facilitating the learning of residual mappings. This design not only mitigates the vanishing gradient issue but also enhances training efficiency (Shan et al., 2024). However, as the network depth increases, ResNet exhibits certain limitations in terms of lightweight design and efficiency. Specifically, ResNet’s primary drawbacks include high computational complexity, redundant parameters, and insufficient lightweight optimization. The computational cost of standard convolution operations in ResNet is significant, particularly in intermediate and deeper modules. For a feature map with input dimensions $H\times W\times C_{\text{in}}$ , the computational complexity of standard convolution is given by Equation 1:


(1) $$FLOPs=H\times W\times C_{in}\times C_{out}\times K^{2}$$

where *K* represents the kernel size, and as the input and output channel counts ( $C_{\text{in}}$ and $C_{\text{out}}$ ) increase, computational complexity escalates rapidly. ResNet, in its pursuit of enhanced model expressiveness, typically stacks multiple standard convolution layers within its modules, resulting in a high parameter count and making deployment on memory-constrained devices challenging.

To address these limitations, we propose an improved version—DIBResNet—designed to significantly reduce computational complexity and parameter count while enhancing model performance. DIBResNet replaces standard convolution operations with DWConv. DWConv consists of two operations: depthwise convolution and pointwise convolution. Depthwise convolution applies a single convolution kernel to each input channel without mixing information between channels, while pointwise convolution performs channel fusion using 1×1 convolutions (Li et al., 2024; Qiu et al., 2024). The computational complexity of depthwise convolution, pointwise convolution, and DWConv is given by Equations 2-4:


(2) $${FLOPs}_{DW}=H\times W\times C_{in}\times K^{2}$$

(3) $${FLOPs}_{PW}=H\times W\times C_{in}\times C_{out}$$

(4) $${FLOPs}_{DS}={FLOPs}_{DW}{+FLOPs}_{PW}$$

Compared to standard convolution, DWConv reduces computational complexity by approximately $1/C_{\text{out}}+1/K^{2}$ times.

Additionally, DIBResNet adopts an inverted bottleneck structure, which enhances feature representation by expanding and then compressing the number of channels. In this structure, the number of channels is first expanded by a factor of e (expansion factor) using pointwise convolution, then features are extracted through depthwise convolution, and finally the channels are reduced to the target number via another pointwise convolution (Shi et al., 2024a). The formula of the inverted bottleneck structure is presented in Equation 5:


(5) $$\text{y}=f_{2}(f_{1}(x))$$

where $f_{1}$ represents the expansion operation and $f_{2}$ represents the compression operation.

For the activation function, we use the Hard-Swish function, whose computational formulation is given by Equation 6:


(6) $$Hard-Swish(x)=x\cdot \frac{ReLU6(x+3)}{6}$$

Compared to ReLU and Swish, Hard-Swish offers higher computational efficiency on hardware while maintaining excellent non-linear representation capabilities.

**Figure 2:**
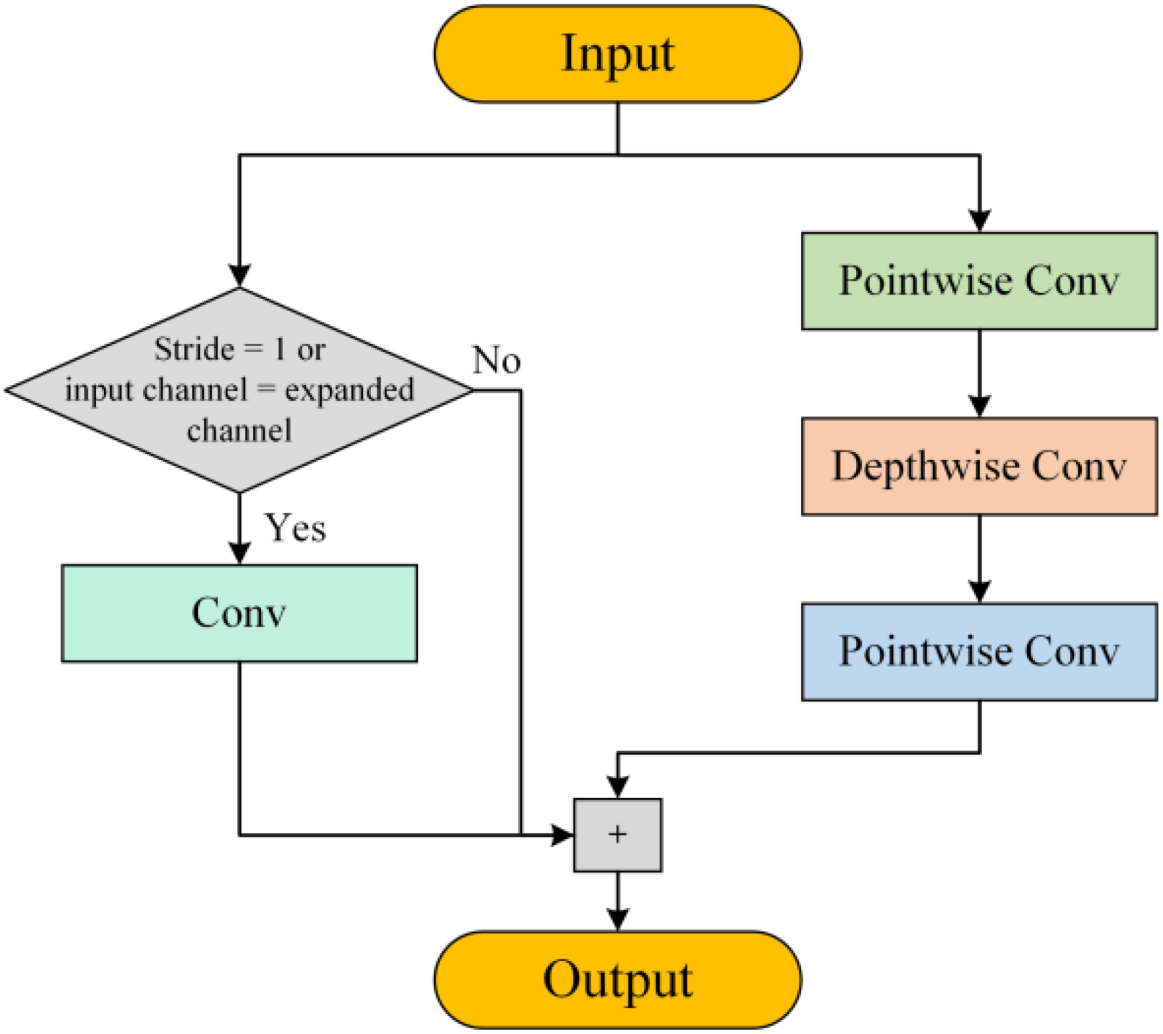
Structural diagram of the DIBResNetBlock.


By balancing feature extraction and computational load, the inverted bottleneck structure and DWConv effectively reduce computational redundancy. The proposed DIBResNet module consists of two components: DIBResNetBlock and DIBResNetLayer. The DIBResNetBlock employs the inverted bottleneck structure, replacing standard convolution with DWConv and incorporating residual connections, its structure is shown in **Figure 2**, and the formula is provided in Equation 7:

(7) $$y=Hard-Swish(f_{3}(f_{2}(f_{1}(x)))+x_{shortcut})$$

where $f_{1},f_{2},f_{3}$ represent pointwise convolution, depthwise convolution, and pointwise convolution, respectively.

**Figure 3:**
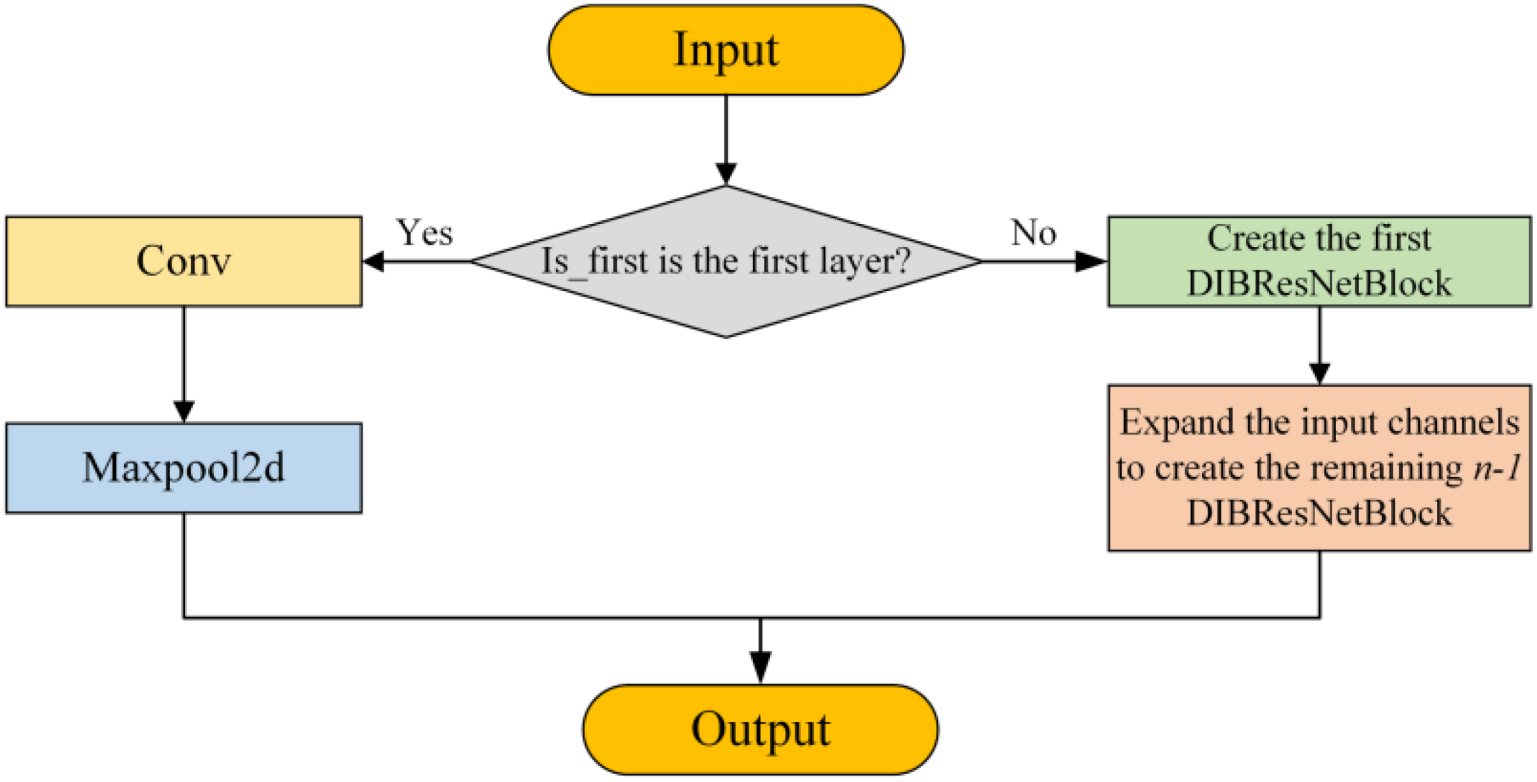
Structural diagram of the DIBResNetLayer.

The DIBResNetLayer stacks multiple DIBResNetBlocks as needed to achieve deep feature extraction. Its structure is illustrated in the **Figure 3**. These improvements allow DIBResNet to significantly reduce computational complexity and parameter count compared to ResNet, while maintaining high accuracy in tasks such as classification and object detection. This makes it particularly suitable for resource-constrained scenarios, including edge devices and mobile applications.

#### 2.2.2 BiFPN

**Figure 4:**
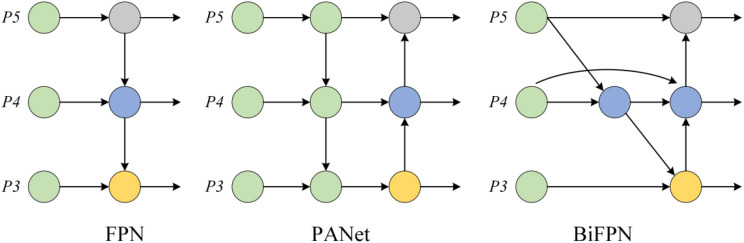
Structure of the BiFPN.

BiFPN is an efficient multi-scale feature fusion module designed to enhance the representation of multi-scale features in deep learning models. Compared to traditional FPN and PANet, BiFPN optimizes feature fusion pathways and computational efficiency. Its core innovation lies in the introduction of learnable feature fusion weights, which dynamically adjust the contributions of different feature maps, enabling the network to aggregate multi-scale information more intelligently (Yu et al., 2024). Additionally, BiFPN employs a simplified bidirectional feature fusion pathway that effectively combines high-level semantic information with low-level detail features. This ensures that key features are captured at all resolutions (Xin et al., 2024). To further enhance computational efficiency, BiFPN applies DWConv after feature fusion, significantly reducing parameter count and computational cost, making it suitable for resource-constrained environments. BiFPN also optimizes network topology by removing redundant edges and nodes, retaining only essential information flow pathways. This reduces computational redundancy while maintaining robust feature representation capabilities. Thanks to these advancements, BiFPN has become a highly efficient module for tasks such as object detection and semantic segmentation, setting a new benchmark for multi-scale feature fusion by balancing computational efficiency and performance. Its structure is illustrated in the **Figure 4**.

#### 2.2.3 GhostConv

**Figure 5:**
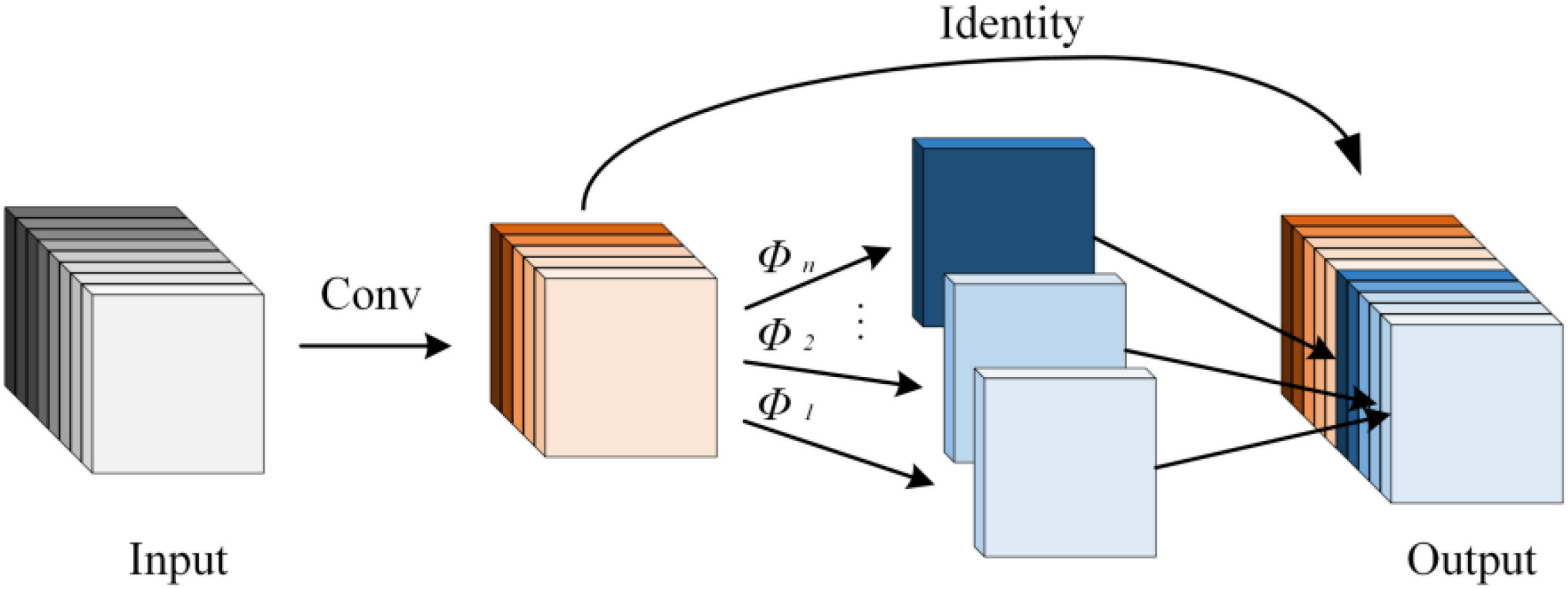
Diagram of the GhostConv structure.


GhostConv is a lightweight convolutional operation designed to reduce computational costs while preserving the model’s expressive capacity by generating redundant feature maps from a smaller subset of primary features. Traditional convolution operations compute complete feature maps, resulting in high computational overhead. GhostConv addresses this issue by first using standard convolution to generate a limited number of primary feature maps, and then applying pointwise convolution to generate additional “redundant” features, thereby reducing computational cost (Zhou et al., 2024). The implementation process consists of two steps: first, standard convolution is used to generate a smaller set of primary feature maps; then, a set of simple linear transformations is applied to produce additional feature maps (Bao et al., 2024). This design significantly reduces computational complexity. The formula for GhostConv is presented in Equation 8, and its structure is illustrated in **Figure 5**. By leveraging this approach, GhostConv can maintain model performance while drastically reducing computational cost, making it an essential module in lightweight network design.

(8) $$Y=[F_{conv}(X);F_{cheap}(F_{conv}(X))]$$

where $F_{conv}$ denotes standard convolution, $F_{cheap}$ represents cheap feature generation operations, and [] indicates the concatenation of feature maps.

#### 2.2.4 Detection head

**Figure 6:**
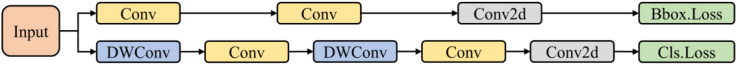
Diagram of the v11detect structure.

This study adopts the v11detect as the head component of the WinterPeachNet model due to its remarkable advantages in object detection tasks. The v11detect inherits key characteristics of the YOLO series while introducing significant improvements and optimizations. The head features decoupled convolutional branches: one branch predicts bounding box regression, and the other focuses on class prediction. This design allows the model to more accurately predict both bounding boxes and classes by enabling each branch to specialize in its specific task. In the v11detect, the convolutional layers for detection and classification are decoupled. The channel count for the feature map in the regression branch is 4×regmax, where regmax denotes the maximum number of predicted bounding boxes. For the classification branch, the channel count corresponds to the number of object classes. Additionally, v11detect employs DWConv in the classification branch, replacing standard convolution to reduce computational cost and improve efficiency. These technical advancements enable the v11detect to achieve high efficiency and accuracy in object detection, providing fast yet precise predictions. The structure of v11detect is shown in **Figure 6**.

#### 2.2.5 WinterPeachNet model

**Figure 7:**
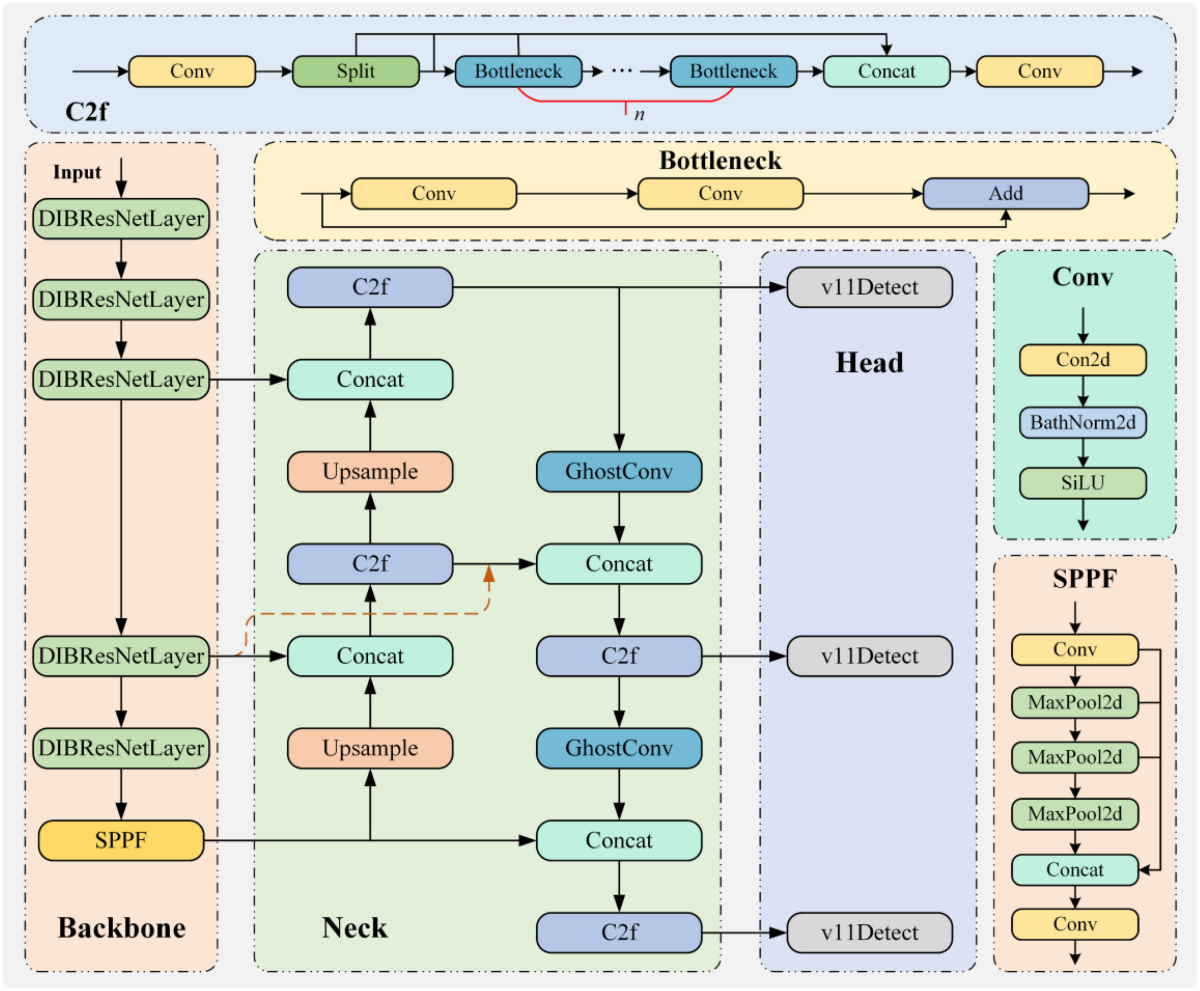
Overall structure of the WinterPeachNet model.

To develop an efficient detection model for winter peach recognition, this study designed a novel recognition model called WinterPeachNet based on the YOLO framework. The backbone network of WinterPeachNet is based on the ResNet architecture and utilizes the DIBResNet structure, which includes DIBResNetBlock and DIBResNetLayer. A DIBResNetLayer consists of multiple DIBResNetBlocks. The DIBResNetBlock employs an inverted bottleneck structure and replaces traditional convolutional operations with DWConv to enhance feature extraction efficiency. This structure first extracts features using DWConv and then fuses the features through pointwise convolution. To address the vanishing gradient problem in deep network training, residual connections are incorporated into the DIBResNetBlock, enabling the network to directly learn residual mappings and facilitating the effective training of deeper layers. In the neck network, the WinterPeachNet model incorporates the BiFPN structure and GhostConv module. BiFPN constructs a bidirectional feature pyramid using top-down and bottom-up information flows, aiding the model in capturing target features at different scales. The GhostConv module increases the depth of convolution by generating virtual convolutional kernels, enhancing feature extraction capability while maintaining computational efficiency. Finally, the model’s head network employs the v11detect, which provides fast and accurate object detection. It processes images in real time and outputs both the locations and classes of the targets. This structural design allows the WinterPeachNet model to excel in winter peach target recognition tasks. It achieves efficient and accurate object detection, offering a novel technological solution for the field of agricultural automation. The overall structure of WinterPeachNet is shown in **Figure 7**.

### 2.3 Evaluation metrics

To evaluate the performance of various models in the task of winter peach object recognition, this study employed precision (P), recall (R), mean average precision (mAP50), and extended mean average precision (mAP50-95) as quantifiable metrics. P measures the proportion of predicted positive samples that are true positives, reflecting the accuracy of the model’s predictions. R measures the proportion of actual positive samples correctly predicted by the model. mAP50 represents the mean average precision at an intersection over union (IoU) threshold of 0.5. IoU is a metric for quantifying the overlap between the predicted and ground-truth bounding boxes, ranging from 0 to 1, with higher values indicating greater overlap. mAP50-95 evaluates the model’s performance across multiple IoU thresholds ranging from 0.5 to 0.95, providing a more comprehensive perspective on detection performance at varying overlap levels. The calculation methods for the evaluation metrics are presented in Equations 9-12:


(9) $$P=\frac{TP}{TP+FP}$$

(10) $$R=\frac{TP}{TP+FN}$$

(11) $$mAP_{50}=\frac{1}{m}\sum_{i=1}^{m}AP_{i}$$

(12) $$mAP_{50-95}=\frac{1}{m}\sum_{i=1}^{m}\frac{1}{10}\sum_{j=1}^{10}AP_{i,j}$$

where TP is the number of true positives, FP is the number of false positives, FN is the number of false negatives, $AP_{i}$ represents the average precision for class *i* at an IoU threshold of 0.5, $AP_{i,j}$ represents the average precision for class *i* at IoU threshold of 0.5+0.05×(j−1) .

## 3 Results

### 3.1 Experimental environment

The experiments were conducted on an Ubuntu 20.04 operating system with Python 3.10 as the programming language and CUDA version 11.8. The deep learning framework employed was PyTorch 2.1.1. The hardware setup included an Intel(R) Xeon(R) Gold 5318Y CPU with a clock speed of 2.10 GHz, an NVIDIA A16 GPU, and 15 GB of memory. In terms of experimental parameter settings, this study adopted the default parameter configuration of the YOLO model. The number of iterations was set to 200, with a batch size of 16. Both the initial and final learning rates were set to 0.01. Additionally, the momentum was set to 0.937, the weight decay coefficient to 0.0005, and the number of warm-up epochs to 3.0. The warm-up momentum was set to 0.8, and the warm-up bias learning rate was set to 0.1. This parameter configuration was chosen based on a comprehensive consideration of model performance and training efficiency, allowing the model to quickly converge in the early stages of training and achieve good performance, thus providing a solid foundation for the experimental research.

### 3.2 Ablation study

Building upon the ResNet architecture, this study proposed an improved model—WinterPeachNet—to enhance the accuracy of winter peach recognition and detection. WinterPeachNet incorporates the DIBResNet structure in the backbone network, integrates the BiFPN structure and GhostConv module in the neck network, and uses the v11detect as the head component to achieve superior recognition performance. The precision results of the ablation study are presented in **Table 1**.

**Table 1:** Precision results of the ablation study.

DIBResNet	GhostConv	BiFPN	v11Detect	P	R	mAP50	mAP50-95	Parameters (M)	GFLOPs
–	–	–	–	0.989	0.978	0.994	0.931	1.722	42.7
✓	–	–	–	0.996	0.984	0.995	0.946	1.097	27.7
✓	✓	–	–	0.992	0.989	0.995	0.946	1.095	27.6
✓	–	✓	–	0.996	0.981	0.995	0.951	1.104	27.9
✓	–	–	✓	0.980	0.989	0.994	0.951	1.089	27.4
✓	✓	✓		0.996	0.985	0.995	0.953	1.102	27.8
✓	✓	✓	✓	0.996	0.996	0.995	0.964	1.094	27.5

The unmodified model has already shown high accuracy in the winter peach object recognition task, with P = 0.989, R = 0.978, mAP50 = 0.994, and mAP50-95 = 0.931. When using DIBResNet as the backbone network, the model’s performance significantly improved, with R increasing by 0.614%, while the number of parameters and GFLOPs were reduced by 36.289% and 35.129%, respectively. Furthermore, this study analyzed the impact of the GhostConv module, BiFPN structure, and v11detect on the model’s performance. The inclusion of the GhostConv module resulted in a 1.125% improvement in R. The integration of the BiFPN structure positively contributed to the model’s recognition accuracy, increasing R by 0.307% and mAP50-95 by 2.148%. Additionally, the application of v11Detect also improved the model’s recognition accuracy, boosting R by 1.125% and mAP50-95 by 2.148%. Notably, despite the improvements made by GhostConv, BiFPN, and v11Detect, these modules did not significantly increase the model’s computational complexity.

When both GhostConv and BiFPN were integrated, the model’s P and R reached 0.996 and 0.985, respectively. mAP50 remained at 0.995, while mAP50-95 increased to 0.953, further proving the importance of the collaborative work of multiple components in improving model performance. Finally, by integrating GhostConv, BiFPN, and v11Detect on top of DIBResNet, the model’s P and R both reached 0.996, with mAP50 at 0.995. Compared to the unmodified model, mAP50-95 increased by 3.545%, and the number of parameters and GFLOPs decreased by 36.492% and 35.597%, respectively. Ablation experiments demonstrate that the WinterPeachNet model, by combining DIBResNet, BiFPN, GhostConv, and v11Detect, significantly improves the recognition accuracy of winter peaches. These improvements not only enhance the model’s P and R but also result in a notable increase in average precision across different IoU thresholds.

### 3.3 Comparative study of different models

**Figure 8:**
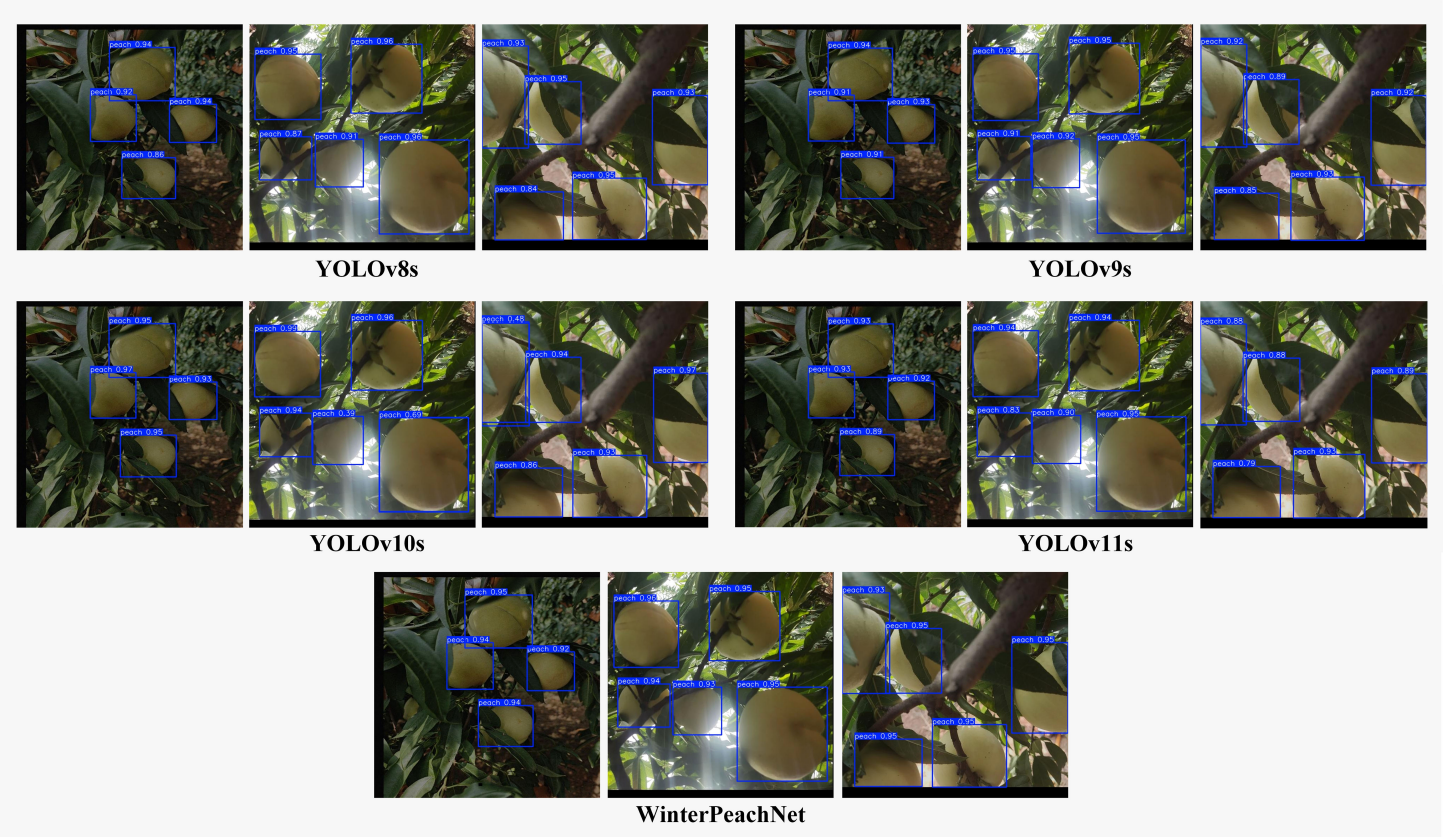
Comparison of detection outcomes for different models.

This study compared the performance of WinterPeachNet with several mainstream YOLO series models. The precision comparison results are summarized in **Table 2**, and detection outcomes for different models are illustrated in **Figure 8**. The results show that WinterPeachNet exhibits significant advantages in precision metrics. It achieved mAP50 = 0.995 and mAP50-95 = 0.964, demonstrating high accuracy in winter peach detection tasks. WinterPeachNet also performed exceptionally well in P and R, both reaching 0.996. This indicates that the model can accurately identify the majority of winter peaches while maintaining a low false positive rate. Although WinterPeachNet has a relatively high parameter count (10.937 M) and computational complexity (GFLOPs = 27.5), its superior accuracy justifies the additional computational resources.

**Table 2:** Results of the comparative study of different models.

Model	P	R	mAP50	mAP50-95	Parameters (M)	GFLOPs
YOLOv8s	0.992	0.978	0.994	0.937	11.126	28.4
YOLOv9s	0.999	0.974	0.994	0.941	7.167	26.7
YOLOv10s	0.973	0.955	0.990	0.941	8.036	24.4
YOLOv11s	0.981	0.985	0.994	0.934	10.711	26.4
WinterPeachNet	0.996	0.996	0.995	0.964	10.937	27.5

By comparison, other YOLO series models, while more lightweight in terms of parameter count and computational complexity, exhibited lower precision. YOLOv8s performs slightly lower than WinterPeachNet in R, with a value of 0.978, while its mAP50 is nearly the same as WinterPeachNet. YOLOv9s and YOLOv10s show slightly lower R, with reductions of 2.209% and 4.117%, respectively, but their mAP50 values are close to that of WinterPeachNet, indicating that they are still competitive in terms of recognition accuracy. YOLOv11s achieves a R of 0.985, but in terms of P and mAP50-95, it still lags behind WinterPeachNet.

In conclusion, WinterPeachNet outperformed other YOLO series models in precision, demonstrating a clear advantage for high-accuracy object detection tasks. While its parameter count and computational complexity are higher, WinterPeachNet is better suited for applications requiring high recognition precision.

## 4 Discussion

This study presents WinterPeachNet, a novel recognition model for winter peach object detection. Built on the ResNet architecture, the model introduces innovations through the integration of the DIBResNet backbone, BiFPN module, GhostConv module, and the v11detect, enhancing the backbone, neck, and head networks. Ablation and comparative experiments validate the effectiveness and superiority of WinterPeachNet.

The results of the ablation experiments reveal the positive impact of each module on the model’s performance. The DIBResNetBlock in the DIBResNet structure, based on inverted bottleneck architecture and DWConv, and incorporating residual connections, plays a key role in improving the model’s P and R. This improvement can be attributed to the reduced computational load of DWConv while capturing features more effectively, and residual connections that alleviate the gradient vanishing problem in deep networks, thus enhancing feature extraction depth and quality. This design significantly improves the applicability of WinterPeachNet in resource-constrained environments. The GhostConv module optimizes the model’s computational efficiency by reducing the number of convolution kernels while maintaining feature extraction capabilities. The BiFPN module, through multi-scale feature fusion, enhances the model’s ability to detect objects of different sizes, improving both R and mAP50-95. The introduction of v11detect, built upon the YOLOv8 detection head, integrates DWConv, making boundary box predictions more precise, as verified in the ablation experiments. Furthermore, the results show that the BiFPN, GhostConv, and v11detect modules do not significantly impact the model’s parameter count or GFLOPs, meaning they improve performance without adding extra computational burden—a critical consideration for resource-limited environments.

In comparison with YOLO series models, although WinterPeachNet performs slightly lower than YOLOv9s in P, it surpasses other YOLO models in all other metrics. In terms of computational cost, WinterPeachNet has fewer parameters and GFLOPs than YOLOv8s, but slightly more than YOLOv9s, YOLOv10s, and YOLOv11s. While not the lightest model, WinterPeachNet’s higher detection accuracy provides a significant advantage in practical applications. By optimizing the network structure and integrating efficient modules, WinterPeachNet achieves high performance in the winter peach detection task. The innovation and optimization of the modules allow the model to maintain high detection accuracy while keeping computational complexity and parameter count relatively low.

Despite its excellent performance in winter peach detection, the generalization ability of the WinterPeachNet model has not been fully validated in other similar fruit or broader object detection tasks. Moreover, the current model design primarily relies on deep learning architecture improvements, and its robustness to issues such as lighting changes and occlusion in complex environments still needs further enhancement. Future research will focus on further optimizing the model structure to improve its generalization and robustness, and validating its performance across a wider range of datasets and application scenarios.

## 5 Conclusion

In this study, we designed and implemented WinterPeachNet, a ResNet-based recognition model tailored for winter peach object detection. By introducing the DIBResNet, BiFPN module, GhostConv module, and v11Detect, this study comprehensively improved the backbone, neck, and head networks of the model. Experimental results demonstrate that WinterPeachNet achieves outstanding performance in winter peach detection, with P = 0.996, R = 0.996, mAP50 = 0.995, and mAP50-95 = 0.964, outperforming several mainstream YOLO-series models. The introduction of DIBResNet significantly improved the depth and quality of feature extraction, effectively reducing the model’s computational complexity. The combined use of the GhostConv and BiFPN modules enhanced the model’s feature extraction capabilities, leading to further improvements in detection accuracy. The inclusion of v11Detect optimized the accuracy of object localization, reducing false positives and false negatives, and played a crucial role in enhancing the overall performance of the model.

The proposed WinterPeachNet model achieves high-precision, low-cost detection of winter peaches. Future research will focus on further optimizing the model’s structure to enhance its object detection capabilities in more complex scenarios. Additionally, the integration of WinterPeachNet with other deep learning technologies will be explored to further improve its performance and efficiency.

## Data Availability

The original contributions presented in the study are included in the article/supplementary material. Further inquiries can be directed to the corresponding author/s.
